# Risk Factors for Acute Kidney Injury in Severe Rhabdomyolysis

**DOI:** 10.1371/journal.pone.0082992

**Published:** 2013-12-18

**Authors:** Eva Rodríguez, María J. Soler, Oana Rap, Clara Barrios, María A. Orfila, Julio Pascual

**Affiliations:** 1 Department of Nephrology, Hospital del Mar, Barcelona, Spain; 2 Nephrology Research Group, Institut Mar d’Investigacions Mediques, Barcelona, Spain; University of Sao Paulo Medical School, Brazil

## Abstract

**Background:**

Acute kidney injury (AKI) is a life-threatening complication of severe rhabdomyolysis. This study was conducted to assess risk factors for AKI and to develop a risk score for early prediction.

**Methods:**

Retrospective observational cohort study with a 9-year follow-up, carried out in an acute-care teaching-affiliated hospital. A total of 126 patients with severe rhabdomyolysis defined as serum creatine kinase (CK) > 5,000 IU/L fulfilled the inclusion criteria. Univariate and logistic regression analyses were performed to determine risk factors for AKI. Based on the values obtained for each variable, a risk score and prognostic probabilities were estimated to establish the risk for developing AKI.

**Results:**

The incidence of AKI was 58%. Death during hospitalization was significantly higher among patients with AKI, compared to patients without AKI (19.2% vs 3.6%, p = 0.008). The following variables were independently associated with AKI: peak CK (odds ratio [OR] 4.9, 95%CI 1.4-16.8), hypoalbuminemia (< 33 mg/dL, [OR 5.1, 95%CI 1.4-17-7]), metabolic acidosis (OR 5.3, 95%CI 1.4-20.3), and decreased prothrombin time (OR 4.4, 95% CI 1.3-14.5). A risk score for AKI was calculated for each patient, with an OR of 1.72 (95%CI 1.45-2.04). The discrimination value of the predictive model was established by means of a ROC curve, with the area under the curve of 0.871 (p<0.001).

**Conclusions:**

The identification of independent factors associated with AKI and a risk score for early prediction of this complication in patients with severe rhabdomyolysis may be useful in clinical practice, particularly to implement early preventive measures.

## Introduction

Rhabdomyolysis, a syndrome characterized by disintegration of skeletal muscle, results in release of muscle cell elements such as myoglobin and creatine kinase (CK) into the blood stream and urine. This syndrome can present as an incidental increase in serum CK levels or as a life-threatening condition with very high CK values [1] causing electrolyte disturbances, acute kidney injury (AKI), and disseminated intravascular coagulation. [2] The pathogenesis of rhabdomyolysis-induced AKI is not fully elucidated, although experimental evidence suggests that intrarenal vasoconstriction, direct and ischemic tubule injury, and tubular obstruction are the main mechanisms involved. [3] One of the key compounds released into the peripheral blood is myoglobin, a 17.8-KdA oxygen carrier similar to hemoglobin, although it contains only one heme moiety. Myoglobin is filtered by the glomeruli and reaches the tubules, where it causes obstruction when interacting with Tamm-Horsfall protein in acidic urine. [2] Myoglobin can also promote intrarenal vasoconstriction by scavenging the vasodilator nitrous oxide from the renal microcirculation. It has been suggested that heme- and free iron-driven hydroxyl radicals are critical mediators of tubule damage owing to the protective effects of deferoxamine and glutathione. More recently, it has been shown that myoglobin itself can exhibit peroxidase-like enzyme activity that leads to uncontrolled oxidation of biomolecules, lipid peroxidation, and the generation of isoprostanes. [4] Other intracellular compounds, such as protons, phosphate, potassium, nucleotides, and precursors of uric acid, are released from the damaged muscles as well, and play an important role in the pathophysiology of rhabdomyolysis-induced AKI. [5], [6].

AKI is the most serious, and even life-threatening, complication of rhabdomyolysis, and is also quite common, representing 7% to 10% of all cases of acute kidney injury in United States [7]–[9] Although the true incidence is difficult to establish owing to varying definitions and clinical scenarios, the reported AKI incidence ranges from 13% to 50%. [9]–[11] Mortality data vary widely according to the study population and setting and the number and severity of coexisting conditions. Among patients in the intensive care unit, mortality has been reported to be 59% when AKI is present and 22% when it is not.^.^ [12]–[13]

This study was conducted to identify and assess the ability of laboratory and clinical parameters to predict the development of AKI in patients with acute severe rhabdomyolysis. A simple risk score was also developed to predict AKI in patients with rhabdomyolysis.

## Materials and Methods

A retrospective study was designed in which a systematic chart review, using the hospital’s coding system, identified all patients admitted to the University Hospital del Mar in Barcelona, Spain, between January 1998 and December 2006 with the International Classification of Diseases (ICD-9) diagnosis of rhabdomyolysis. Patients were included in the study if they were at least 18 years of age and had a serum CK level ≥ 5,000 IU/L within the first 48 hours of hospital admission. The aim was to identify patients at high risk of AKI, because previous studies had shown that more than 50% of patients with a CK level > 5,000 IU/L developed AKI. [14] Patients with chronic kidney disease and acute coronary disease were excluded.

This study adhered to the Principles of Helsinki Declaration, and the hospital’s Ethics Committee (CEIC-IMAS) approved the study protocol. Written informed consent was not required because of the non-intervention and retrospective chart review characteristics of the study.

### Data collection and study design

The main etiology of rhabdomyolysis was determined by chart review; if was not possible to select only one etiology, the case was classified as “Other.” Demographic data were collected, along with past medical history of diabetes mellitus, dyslipidemia, chronic kidney disease, human immunodeficiency virus [HIV] infection, acquired immunodeficiency syndrome [AIDS], hepatitis C virus infection [HCV], hepatitis B virus infection [HBV], ischemic heart disease, and stroke. Medications, including statins, used at the time of admission were recorded, along with history of recent physical exertion, traumatisms, seizures, immobilization, infectious disease, and consumption of alcohol or other drugs. Presenting symptoms including fever (body temperature > 37.5°C), hypertension (systolic blood pressure [SBP] > 140 mmHg and/or diastolic blood pressure [DBP] > 90 mmHg) or hypotension (SBP <90 mmHg and/or DBP <60 mmHg), oliguria (diuresis <400 mL/24 h), and compartment syndrome were recorded.

Laboratory data on admission included blood urea nitrogen, serum creatinine, potassium, calcium, prothrombin time, acid-base equilibrium, lactate dehydrogenase (LDH) and liver function parameters (albumin, alanine aminotransferase [ALT], aspartate aminotransferase [AST], gamma-glutamyl transferase [GGT] and prothrombin time). The highest value of CK during the first 48 hours of hospital admission was analyzed. The time-frame was set at 48 hours to avoid the possibility that an interrecurrent process later in the hospitalization could produce a misclassification of the rhabdomyolysis etiology.

Severe rhabdomyolysis was defined according to serum CK levels > 5,000 IU/L, hypoalbuminemia (serum albumin <33 mg/dL), hyperkalemia (serum potassium > 5.5 mEq/L), metabolic acidosis (pH<7.35), hypocalcemia (serum calcium <8.5 mg/dL), and decreased prothrombin time (< 82%). Normal levels for AST were 17–41 UI/L, ALT 10–35 UI/L and GGT 8–61 UI/L.

AKI was defined according to the ADQI (Acute Dialysis Quality Initiative) criteria (RIFLE classification). [15], [16] Briefly, patients were classified into the “risk” category if serum creatinine increased 1.5-fold or glomerular filtration rate (GFR) decreased > 25%, “injury” if serum creatinine increased 2-fold or GFR decreased > 50%, and “failure” if serum creatinine increased 3-fold or GFR decreased > 75%. The outcome criteria of loss of renal function and end-stage renal disease (ESRD) were defined by the duration of the acute kidney injury.

Baseline serum creatinine values were assessed by two methods. We checked to see if the patient had a normal kidney function blood test at least 3 months before the rhabdomyolysis episode, either in our center or in the relevant basic health area. We also estimated kidney function using the Modification of Diet in Renal Disease (MDRD) equation as recommended by the ADQI Working Group, assuming a lower limit of normal baseline GFR of 75 mL/min, similar to previous studies. [17], [19] For analysis, patients were assigned to their worst RIFLE category according to serum creatinine criteria during the first 48 hours after admission. Patients were stratified according to the presence or absence of AKI.

Serum and urinary myoglobin are not routinely assessed at our site. Because of the retrospective design of the study, most of these data were missing and the variables were not included in the study.

### Statistical analysis

Data are presented as mean (± standard deviation), median (range), absolute numbers or percentages. Differences in descriptive variables between patients with or without AKI were assessed using the chi squared test. Student *t* test was used to compare mean values between the study groups. A logistic regression model with a backwards stepwise selection of variables was used to determine independent variables associated with AKI. Data are expressed as odds ratio (ORs) and 95% confidence intervals (CIs). The predictive values obtained in the logistic regression analysis were used to calculate the individual risk of having AKI according to the following equation in which _0 was the constant of the model, _1 to _p were the regression coefficients of the independent variables, and xli to xpi were the values of the variable for a particular patient.
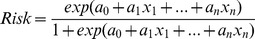



This method has been previously described in detail. [20].

Binary logistic regression was used to calculate the prognostic probability of developing AKI, based on the risk factors present in the setting of rhabdomyolysis. Measures of calibration (Hosmer-Lemeshow statistics) and discrimination (ROC statistic) were calculated to evaluate the overall performance of the predictive model. All calculations were performed using the SPSS software package (v15.0, SPSS Inc., Chicago, IL). Statistical significance was set at p<0.05.

## Results

### Study population

During a follow-up of 9 years, 138 patients admitted to our hospital received a diagnosis of severe rhabdomyolysis. Twelve patients were excluded because of previous history of chronic kidney disease. All of the 126 patients included in the analysis were Caucasian; the mean age was 54±20 years, and 74% were men. The median CK level was 12,750 IU/L. Baseline characteristics of the study population are shown in Table 1. The most frequent cause of rhabdomyolysis was prolonged immobilization due to consumption of drug abuse (27.8%). Heroin was the most frequently abused drug (24%), followed by cocaine (22.4%), narcotics (19.8%), alcohol (13.5%), and smart drugs (5.6%). Pneumonia was the main cause (83%) of rhabdomyolysis secondary to infectious diseases in non-septic patients (19.8% of the sample) (Table 1).

**Table 1:** Characteristics of 126 patients with severe rhabdomyolysis^a^.

Demographic and clinical characteristics	No. (%)
Age, years, mean ± SD	54±20
Gender (male/female)	93 (74)/33 (26)
Smoking	66 (52.4)
Illicit drug use	45 (35.7)
Diabetes mellitus	11 (14)
Arterial hypertension	34 (27.4)
Hyperlipidemia	12 (9.5)
Stroke	6 (4.8)
Ischemic heart disease	4 (3.2)
Positive serological tests for infection: HIV/, HCV/and HBV	23%/28.6%/5%
**Etiology of rhabdomyolysis**	%
Immobilization due to illicit drugs abuse	27.8
Infectious disease	19.8
Traumatism	7.1
Seizures	7.1
Stroke	4.8
Surgery	3.2
Other	30.2

Data as number and percentages in parenthesis unless otherwise stated.

### Acute kidney injury

The mean serum creatinine on admission was 2.5 mg/dl ± 2.2 mg/dl (range 0.5–11 mg/dl). Seventy-three patients developed AKI, 59 of them at hospital admission and 14 patients within the first 48 hours after admission. Twelve patients (9.5%) needed renal replacement therapy (hemodialysis, n = 10; peritoneal dialysis, n = 2). Dialysis was initiated at a mean of 2 days from admission (range 1–4 days), and was maintained for 8.3 (2-21) days. Fifty-nine patients who survived AKI were discharged from the hospital with a fully recovered kidney function.

In relation to RIFLE classification, 14 patients (19.2%) were included in the “risk” group, 18 (24.7%) in the “injury” group and 41 (56.2%) in the “failure” group. Loss of renal function or ESRD was not observed.

### Mortality

The in-hospital mortality rate was 14% (n = 18). Mortality was significantly higher among patients who developed AKI (n = 14) than in patients without AKI (n = 4) (19.2% *vs* 3.6%, p = 0.008). Causes of death were septic shock (9.4%), intoxication or adverse drug effects (2.2%), disseminated intravascular coagulation (0.7%), and fatal arrhythmia (0.7%).

### Clinical characteristics and laboratory data

Intravenous heroin use was significantly more frequent in patients with AKI than in those without AKI (43.8% *vs* 24.5%, p = 0.026), and a higher percentage of patients with AKI had HCV infection (35.6% *vs* 18.9%, p = 0.004). There were no differences between patients with and without AKI according to gender, age, history of diabetes, ischemic heart disease, hypertension, HBV, and HIV infection.

### Risk factors for AKI

The presence of the following variables on admission was significantly associated with AKI: hypoalbuminemia, hyperkalemia, ALT > 259 IU/L, AST > 95 IU/L, hypocalcemia, metabolic acidosis, decreased prothrombin time (PT), and diagnosis of disseminated intravascular coagulation (Table 2). The serum CK level during the first 48 hours of admission was also related to AKI (Table 2).

**Table 2:** Comparison of clinical characteristics and peak laboratory values between patients with severe rhabdomyolysis with or without AKI.

Variable	AKI (n = 73) No. (%)	Non-AKI (n = 43) No. (%)	P value
Illicit drugs	32 (43.8%)	13 (24.5%)	0.026
HCV infection	26 (35.6%)	10 (18.9%)	0.04
Disseminated intravascular coagulation	17 (23.3)	1 (1.9)	<0.001
Albumin (< 33 g/L)	48 (75)	15 (37.5)	<0.001
Potassium (≥ 5.5 mEq/L)	15 (20.5)	2 (3.8)	0.007
ALT (≥259 IU/L)	44 (62.9)	17 (32.7)	0.001
AST (≥95 IU/L)	35 (59.3)	12 (31.6)	0.008
GGT (≥34 IU/L)	37 (63.8)	9 (25.7)	<0.001
Calcium (<8.5 mg/dL)	39 (58.2)	12(27.9)	0.002
Metabolic acidosis	41 (56.2)	9 (17.3)	<0.001
Prothrombin time (<82%)	47 (65.3)	15 (28.8)	<0.001
Peak CK (>12,750 IU/L)	46 (63.9)	16 (30.2)	<0.001

HCV: Hepatitis C virus ALT: alanine aminimotransferase; AST: aspartate aminotransferase; GGT: gamma glutamyl transferase; CK: creatine phosphokinase.

By logistic regression analysis (Table 3), variables independently associated with AKI included serum CK (OR 4.9, 95% CI 1.4-16.8), hypoalbuminemia (HA) (OR 5.1, 95% CI 1.4-17-7), metabolic acidosis (MA) (OR 5.3, 95% CI 1.4-20.3), and decreased PT (HT) (OR 4.4, 95% CI 1.3-14.5). A risk score for the prediction of AKI was calculated for each patient using the following equation:

**Table 3:** Variables associated with AKI in patients with severe rhabdomyolysis. Results of logistic regression analysis.

Variable	Odds ratio	95% confidence interval	P value
Albumin (< 33 g/L)	5.1	1.4 - 17.7	<0.01
Metabolic acidosis	5.3	1.4 - 20.3	<0.01
Prothrombin time (<82%)	4.3	1.3 - 14.5	<0.01
Peak CK (>12,750 IU/L)	4.9	1.4 - 16.8	<0.01

CK: creatine phosphokinase.



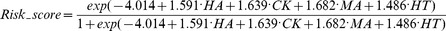



Binary variables were coded as follows: CK level ≤ 12,750 UI/L = 0, CK level > 12,750 UI/L = 1, albumin > 33 mg/dL = 0, albumin ≤ 33 g/L  = 1; venous pH > 7.35 = 0, venous pH ≤ 7.35 = 1; PT > 82% = 0, PT ≤ 82% = 1.

**Figure 1:**
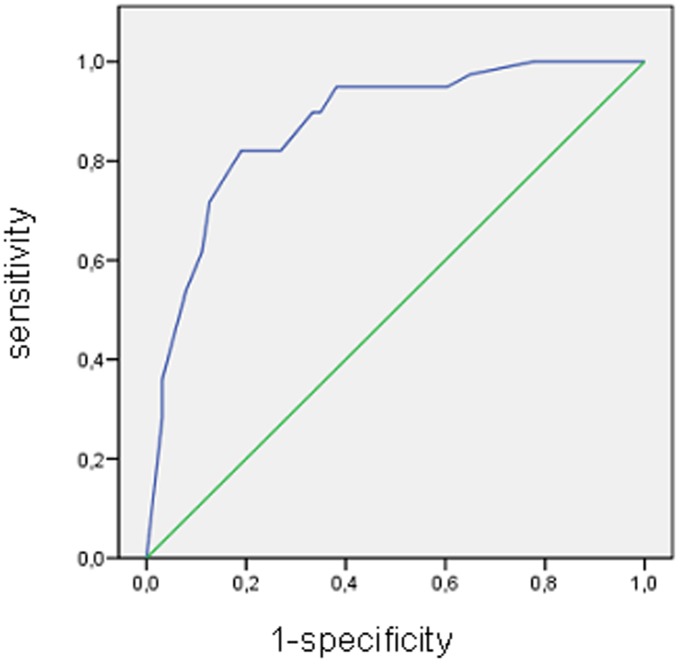
Receiver operating characteristic (ROC) curve for the predictive model (risk score equation) for AKI in severe rhabdomyolysis.

The OR of this score for predicting AKI was 1.72 (95% CI 1.45-2.04). The discrimination value of the predictive model was established by means of a ROC curve, with area under the curve (AUC) of 0.871 (p<0.001); Hosmer-Lemeshow goodness-of-fit-statistic was 2.404 (Figure 1).

**Figure 2:**
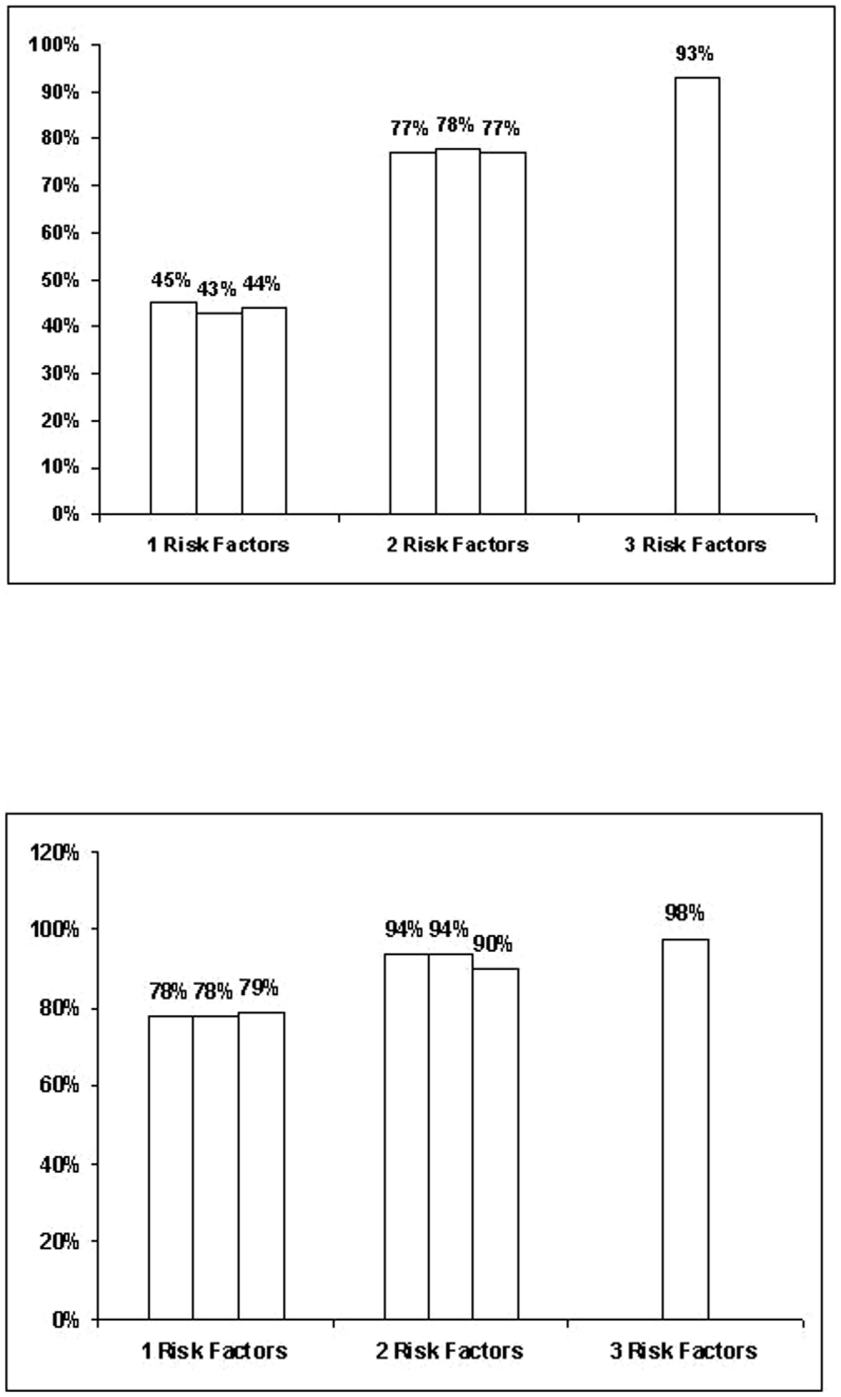
Prognostic probability of developing AKI in severe rhabdomyolysis according to risk factors at hospital admission. Percentages indicate the proportion of patients developing AKI if creatine phosphokinase (CK) is above or below 1,2750 IU/L and additional risk factors are present (e.g., a patient with hypoalbuminemia [< 33 g/l], metabolic acidosis, PT <82% and peak CK >1,2750 IU/L has a 98% likelihood of developing AKI). Figure 2A: CPK ≤12,750 IU/L. Figure 2B: CPK >12,750 IU/L.

Binary logistic regression was used to calculate the predicted probability of developing AKI. Patients presenting with CK levels >12,750 IU/L, hypoalbuminemia, metabolic acidosis, and decreased PT<82% had a 98% probability of developing AKI. In contrast, patients with CK levels ≤ 12,750 IU/L, normal serum albumin levels, normal acid base equilibrium and prothrombin time >82% had a low probability (16%) of developing AKI (Figure 2).

## Discussion

The identification of patients with severe rhabdomyolysis who are at risk for AKI remains critical because they are exposed to increased mortality and morbidity that could be overcome by appropriate preventive measures, such as aggressive early fluid repletion. [21], [22] To our knowledge, no previous study has aimed at validating a risk score and a predictive model to estimate the risk of AKI in patients with severe rhabdomyolysis. This approach provides a novel tool to establish an early diagnosis of AKI and initiate individualized treatment without delay. Gabow et al. [5] reported a formula for predicting patients with rhabdomyolysis at high risk of developing AKI, based on discriminant analysis. This formula differentiated between “high” and “low” risk groups and was intended for use with patients having serum creatinine values <3 mg/dL. Hatamizadech et al. [23] developed a score system based on the Bam earthquake, but the definition of AKI (serum creatinine > 1.6 mg/dL) can be questioned. They reported that both uric acid and LDH are risk factors for AKI and are closely related to the underlying pathophysiology of AKI. In our study, logistic regression analysis was used to determine the AKI risk for each individual patient.

In the present study, a series of 126 patients with severe rhabdomyolysis (CK > 5,000 IU/L) admitted to a teaching hospital in Barcelona (Spain) during a 9-year period were assessed. AKI occurred in 58% of the patients, which in turn was associated with five times higher in-hospital mortality compared to patients with rhabdomyolysis but without AKI. These findings are consistent with a previous study in an intensive care unit, in which mortality was nearly three times higher among patients with acute renal failure due to rhabdomyolysis than that of patients without renal failure. [12].

The most common etiology of rhabdomyolysis in our population was intravenoous drug use (mainly heroin). In contrast, in the United States, where about 26,000 cases of rhabdomyolysis are reported annually in the National Hospital Discharged Patients Database, the main underlying cause of rhabdomyolysis is alcohol intoxication, followed by illicit drug use. [21], [22].

Among other analytical parameters included in the multivariate analysis, CK levels, hypoalbuminemia, decreased PT, and metabolic acidosis on admission were independent risk factors for AKI. These laboratory data are related to the extent of muscle injury (CK levels, metabolic acidosis) and the general condition prior to the index event (hypoalbuminemia and decreased PT). Our results showed high CK values (median 12,750 IU/L) are related with AKI, although it has been reported that the risk of AKI is usually low when CK values on admission are lower than 15,000 to 20,000 IU/L. [23] In agreement with the present findings, some studies in patients with rhabdomyolysis have found that the peak CK level is an independent predictor for the development of AKI. [22] Meijer et al. [12] showed that serum CK levels are correlated with AKI in patients with severe rhabdomyolysis, and kinetic analysis has shown that CK values declined faster in patients without AKI. [12]

Based on the aforementioned laboratory results, a model to calculate the risk of developing AKI in individual patients was designed using a methodology already reported and validated in other medical conditions. [20] In 2004, a consensus definition and classification scheme for AKI, the RIFLE criteria, was developed. [15] These criteria have now been extensively evaluated across a range of clinical settings and have been shown to have predictive ability, robustness, and clinical relevance. [17]–[19] A recent study used RIFLE criteria to stratify the severity of AKI and to predict prognosis in patients with acute rhabdomyolysis at the emergency setting. [24] The RIFLE categories correlated significantly with known markers of rhabdomyolysis and AKI. These categories also predicted length of stay, need for dialysis procedures, renal morbidity, and timing of recovery. In our study, patients who developed AKI were classified according to these ADQI stages but statistical differences between the different ADQI stages were not observed (data not shown). The lack of correlation was probably related to the fact that most patients were classified into the “failure” stage.

The mortality rate was 13%. This reinforces the poor prognosis of this disease, a serious condition that often leads to acute kidney failure and death. Despite this high in-hospital mortality rate, long-term renal outcomes remain favorable upon recovery because survivors who were discharged from the hospital with a fully recovered renal function no longer require chronic renal replacement therapy. These data are in agreement with previous reports. [13]

Our study has some limitations. The retrospective design precluded a more comprehensive assessment of each case. In addition, a larger patient sample could perhaps have made our logistic regression model more robust. Internal validation of the risk score formula developed in the study is needed in an additional prospective population of patients with rhabdomyolysis. External validation of the predictive model and score in other medical conditions associated with high risk of AKI also could be of interest.

In summary, specific laboratory test results and two models (the risk score formula and the prognostic probability) predicted AKI in patients with severe rhabdomyolysis. This approach may help to identify those patients who are at high risk for developing AKI, thus alerting the clinician to the need for aggressive medical management. Currently, the main step in managing rhabdomyolysis remains the early use of aggressive fluid replacement therapy, although the composition of fluids used for repletion remains controversial. [25]–[29] Patients in whom AKI developed had a longer delay in receiving fluid therapy than did patients in whom AKI did not develop. [28] Our findings may help to anticipate adequate fluid therapy and prevent kidney injury.
